# Risk Factors of Seizure in Childhood Shigellosis

**DOI:** 10.22037/ijcn.v18i2.43652

**Published:** 2024-03-12

**Authors:** Abolfazl MAHYAR, Shifteh MAHYAR, Sonia OVEISI, Bahman KHAJEH, Khatereh KHAMENEHPOUR, Victoria CHEGINI, Reza DALIRANI, Mojgan ENADI, Shiva ESMAEILI

**Affiliations:** 1 Qazvin Children Hospital, Qazvin University of Medical Sciences, Qazvin, Iran.; 2 Pediatric Nephrology Department, Mofid Children’s Hospital, Faculty of Medicine, Shahid Beheshti University of Medical Sciences, Tehran, Iran

**Keywords:** Children, Seizure, Shigellosis

## Abstract

**Objectives:**

Shigellosis is one of the common causes of bacterial diarrhea in children. Seizures are common in shigellosis. It is essential to identify the risk factors of seizure in this disease.

**Materials & Methods:**

This study was conducted on 224 children with shigellosis. The patients were divided into: With (case groups = 63 cases) and without seizures (control group = 161 cases). Groups were compared regarding different variables such as age, gender, clinical symptoms, and laboratory findings. Data analysis was done using statistical tests and SPSS software. Logistic regression analysis was used to determine the risk factors of seizures.

**Results:**

Out of 224 cases of children with shigellosis, 107 (47.8%) were male and 117 (52.2%) female. Significant differences were observed between the two groups in terms of age, history of febrile convulsions, frequency of bloody diarrhea, frequency of fever, duration of diarrhea before hospitalization, abdominal pain, increase in BUN, hyponatremia, hypocalcemia, and red blood cell count in stool (P<0.05). Logistic regression analysis showed that a history of febrile seizure, fever, and hyponatremia are the risk factors for seizures in shigellosis.

**Conclusion:**

This study concluded that a history of febrile seizure, fever, and hyponatremia are risk factors for seizure in childhood shigellosis, thus rapid diagnosis and treatment of childhood shigellosis with risk factors is very important.

## Introduction

Shigellosis is an infectious disease caused by the invasion of different Shigella species into the epithelium covering the terminal ileum, colon, and rectum (1-2). This disease is one of the important causes of dysentery in children. Shigella is a gram-negative bacterium, comprising four types: S. flexneri, S.sonnei, S. boydii, and S. dysenteriae (2). Shigella dysentery was first reported in Japan by Kiyoshi Shiga in 1897. After that, Shigella flexneri in 1899, Shigella sonnei in 1906 by Carl Olaf Sonne, and Shigella boydii in 1921 were identified (3). The disease occurs globally and affects all ages, but it is more prevalent in developing countries and among children aged less than 5 years old (1). According to the Seed report, 80 to 165 million people in the world are infected with shigellosis every year and 600,000 people die due to this disease. Most of these deaths occur in developing countries with inadequate public health (2). Clinical symptoms of shigellosis vary and include intestinal and extraintestinal manifestations (2, 4-5). One of the common extraintestinal manifestations is neurological manifestations, which can manifest in various forms, including seizures (2). The fatal form of Shigella encephalopathy is called Ekiri syndrome, associated with high mortality (2,4-5) The exact pathophysiology of neurological manifestations is not known. Studies conducted in the field of identifying the risk factors for seizures in children with shigellosis are limited (4, 5). Ashkenazi et al. believe that factors apart from Shiga toxin are involved in neurological mechanisms (6). Khan et al. reported that Shigella encephalopathy is more common in Shigellosis (4). Afroze et al.’s study showed that hyponatremia, short duration of diarrhea before hospitalization, and sepsis are risk factors of disease exacerbation and death of patients (5). According to the above-mentioned content, this study aimed to identify the risk factors of seizures in children with shigellosis.

## Materials & Methods

This cross-sectional study was conducted on 224 children with shigellosis hospitalized at Qazvin Children’s Hospital for six years (2016-2021), the only teaching hospital in Qazvin province affiliated with Qazvin University of Medical Sciences (Qazvin, Iran). The children were between one month and 12 years old. Sampling was based on census. Children with the following conditions were included (inclusion criteria): 1) having symptoms of dysentery such as fever, abdominal pain, mucus diarrhea with or without bloody stool and with or without vomiting, 2) presence of white blood cells with or without red blood cells in direct stool test, 3) Positive stool culture for Shigella organism, and 4) Conducting antibiotic sensitivity and resistance tests on all samples.

 Children with 1) Abnormal direct stool test and normal stool culture and 2) Failure to perform sensitivity and resistance tests were excluded. Children with shigellosis were divided into two groups: With (case group) and without seizures (control group). Several factors, including age, gender, body mass index (BMI), fever, length of diarrhea, vomiting duration, clinical symptoms, laboratory findings, and stool culture sensitivity results, were analyzed between the two groups. Weight, height, and BMI z-score were measured as a standard method (2, 7). All laboratory test was done as standard method in the laboratory department of Qazvin Children’s Hospital. First, the required information was extracted from the patients’ files and then recorded in the information form. The chi-square test was used to compare qualitative variables. Besides, t-student test (mean ± SD) and Wilcoxon test [median (IQR) Interquartile Range] were used for quantitative variables. The logistic regression analysis was used to determine seizure risk factors. Statistical analyzes were performed using SPSS for Windows 16.0 software (SPSS Inc., Chicago, IL). P-value<0.05 was considered statistically significant

## Results

Out of 224 cases of children with shigellosis, 107 (47.8%) were male and 117 (52.2%) female. The minimum and maximum ages of the patients were one and 144 months, respectively, with a median (IQR) of 60 ± 48 months. The abundance of Shigella species included sonnei 123 cases (50.5%), flexneri 91 cases (45.1%), boydii nine cases (4%), and dysentria one case (0.4%). The highest antibiotic sensitivity and resistance were to ciprofloxacin (171 cases) and CO-TMX (203 cases), respectively. Out of 224 children with shigellosis, 63 children had seizures (case group) and 161 children without seizures (control group). The mean ± SD of the age in the case group was 51 ± 40 months and in the control group 60 ± 51 months (P<0.05). The ratio of males to females in the case group was 29/34 and in the control group was 78/83(P>0.05). A significant difference was observed between the two groups in terms of history of febrile seizure, frequency of bloody diarrhea, frequency of fever, duration of diarrhea before hospitalization, abdominal pain, increase in BUN, hyponatremia, hypocalcemia, and the number of stool red blood cells (Table 1-3). P<0.05). No significant difference was found between the two groups in terms of the frequency of different species of Shigella (P>0.05). The comparison of antibiotic resistance showed that a significant difference was found between the two groups in terms of ciprofloxacin and nalidixic acid (Table 4). ) (P<0.05). No significant difference existed between two groups regarding multi-drug resistance pattern (P>0.05). Logistic regression analysis after adjustment of potential confounding factors showed that the history of febrile seizure, fever, and hyponatremia are independent risk factors for seizure in shigellosis (P<0.05) (Table 5). Moreover, indicatively, children with Shigella seizure have significantly fewer cases of ESR more than 30 mm/h, red blood cells more than 10/HPF, bloody diarrhea, and abdominal pain(P<0.05).

**Table 1:** Comparison of demographic pattern between Shigella children with (case group) and without seizures (control group)

P- value	Unadjusted 95% CI	Unadjusted OR	Control group (n=161)	Case group (n=63)	Characteristics
0.93	0.9-1.1	1	43(26.7%)	19(30.2)	Month admission1
0.59	0.71-1.41	1	67(41.6)	32(50.8)	Season admission1
0.53	0.73-1.48	1.04	35(27.8) 42(33.3) 40(31.7) 7(5.6) 2(1.6)	14(30.4) 10(21.7) 19(41.3) 3(6.5) 0(0)	BMI Z-Score1: Severe Underweight Underweight Normal weight Overweight Obesity
0.53	0.28-1.9	0.73	20(12.5)	6(9.5) 1	Use of antibiotic before admission1
0.00	2.39-165	19.87	1(0.6)	7(11.1) 1	History of previous febrile seizure1

Frequency (Chi-square test) 1

**Table 2:** Comparison of clinical characteristics between Shigella children with (case group) and without seizures (control group)

P- value	Unadjusted 95% CI	Unadjusted OR	Control group (n=161)	Case group (n=63)	Characteristics
-	-	-	161(100)	63(100)	Diarrhea1
0.001	0.1-0.59	0.25	53(32.9)	7(11.1)	Bloody diarrhea1
0.18	0.32-1.24	0.63	53(32.9)	15(23.8)	Vomiting1
0.003	1.45-7.38	3.28	109(67.7)	55(87.3)	Fever 1
0.000	0.14-0.57	0.28	60(43.5) 78(56.5)	38(73.1) 14(26.9)	Duration of diarrhea before admission1 <24 hour >24 hour
0.45	0,05-3.8	0.43	43(86) 7(14)	14(93.3) 1(6.7)	Duration of vomiting before admission1 <24 hour >24 hour
0.02	0.07-0.89	0.26	26(16.1)	3(4.8)	Abdominal pain1
0.54	0.67-1.68	1.06	125(78.6) 19(11.9) 15(9.4)	47(74.6) 11(17.5) 5(7.9)	Dehydration1: Mild Moderate Severe
0.84	0.11-14.3	1.28	2(1.2)	1(1.6)	Hemolytic uremic syndrome( HUS) 1

Frequency (Chi-square test) 1

**Table 3:** Comparison of laboratory profile between Shigella children with (case group) and without seizures (control group)

P- value	Unadjusted 95% CI	Unadjusted OR	Control group (n=161)	Case group (n=63)	Characteristics
0.68	0.47-3.14	1.21	15(9.3)	7(11.1)	Leukocytosis(>15000/ µL) 1
0.73	0.56-2.24	1.12	35(21.7)	15(23.8)	Leucopneia(<5000/ µL) 1
0.1	0.89-2.95	1.63	53(32.9)	28(44.4)	Bandemia( >5%)1
0.58	0.43-4.3	1.38	14(9.2) 139(93.8)	4(6.8) 55(93.2)	Hemoglobin(gr/dL) 1 <11 >11
0.4	0.47-6.3	1.73	13(8.5) 140(91.5)	3(5.1) 56(94.4)	Hematocrit1 <33 >33
0.65	0.42-3.94	1.29	10(6.3)	5(7.9)	Anemia (<11gr/dL) 1
0.62	0.28-10.5	1.71	3(1.9)	2(3.2)	Thrombocytosis (>450000/µL) 1
0.8	0.22-7.16	1.27	4(2.5)	2(3.2)	Thrombocytopneia(<150000/µL) 1
1	0.45-2.4	1.05	137(85.1)	54(85.7)	CRP >6/(mg/L) 1
0.00	0.08-0.5	0.2	55(34.2)	6(9.5)	ESR >30 (mm/hr) 1
0.03	-	0	11(6.8)	0(0)	Increased BUN1
0.88	0.37-2.34	0.93	19(11.8)	7(11.1)	Increaed Creatinine1
0.03	1.03-3.59	1.92	39(24.2)	24(38.1)	Hyponatremia (<135 meq/L) 1
0.28	-	-	0(0)	1(1.6)	Hypernatremia(>150meq/L) 1
0.85	0.48-2.4	1.07	24(14.9)	10(15.9)	Hypokalemia(>3.5meq/L) 1
0.19	0.46-58	5.24	1(0.6)	2(3.2)	Hyperkalemia(>5.5meq/L) 1
0.13	0.64-24.3	3.97	2(1.2)	3(4.8)	Hypoglycemia(<60mg/dL) 1
0.09	0.91-3.29	1.73	36(22.4)	21(33.3)	Hyperglycemia(>140mg/dL) 1
0.02	-	-	0(0)	3(4.8)	Hypocalcemia (7.5mg/dL) 1
1	0.08-8.32	0.84	3(1.9)	1(1.6)	Metabolic acidosis1
0.78	0.63-1.96	1.11	7(4.4) 29(18.1) 124(77.5)	3(4.8) 9(14.3) 51(81)	Stool leucocyte(/ HPF) 1: <10 11-50 >50
0.03	0.53-1.03	0.73	40(25) 44(27.5) 76(47.5)	26(41.9) 10(16.1) 26(41.9)	Stool RBC(/ HPF) 1: <10 11-50 >50

Frequency (Chi-square test) 1

**Table 4:** Comparison of Shigella antibiotic resistance pattern between Shigella children with (case group) and without seizures (control group)

P- value	Unadjusted 95% CI	Unadjusted OR	Control group (n=161)%	Case group (n=63)%	Shigella antibiotic resistance
0.56	0.63-2.32	1.21	40(24.8)	18(28.6)	Ampicillin
0.64	0.44-3.65	1.28	145(90.1)	58(92.1)	C0-TMX
0.49	0.66-2.31	1.24	102(63.4)	43(68.3)	Ceftriaxone
0.1	0.82-5.8	2.19	10(6.2)	8(12.7)	Cefotaxime
0.97	0.52-1.82	0.97	52(32.3)	20(31.7)	Ceftazidime
0.19	0.46-58.9	5.24	1(0.6)	2(3.2)	Ceftizoxime
0.69	0.6-2.13	1.13	108(67.1)	44(69.8)	Cefixime
0.039	0.2-.97	0.44	44(27.3)	9(14.3)	Ciprofloxacine
0.68	0.069-5.77	0.63	4(2.5)	1(1.6)	Imepneum
-	-	-	0	0	Merupneum
1	0.15-41	2.58	1(0.6)	1(1.6)	Amikacin
0.39	0.47-6.4	1.75	6(3.7)	4(6.3)	Gentamycin
0.022	1.1-3.7	2.02	80(49.7)	42(66.7)	Nalidixic acid

Frequency (Chi-square test) 1

**Table 5:** Logistic regression analysis for detection of independent risk factors in children with childhood Shigella seizure

p value	Adjusted 95% CI	Adjusted OR	Characteristics
0.018	1.57-130	14.33	History of previous febrile seizure
0.006	1.39-7.42	3.21	Fever
0.023	1.13-5.66	2.54	Hyponatremia
0.001	0.069-0.49	0.18	ESR
0.032	0.39-0.95	0.61	Stool RBC
0.002	0.1-0.59	0.24	Bloody diarrhea
0.032	0.069-0.88	0.25	Abdominal pain
0.008	0.126-0.73	0.3	Duration of diarrhea before admission
0.22	0.31-1.31	0.64	Duration of vomiting before admission
0.96	0.61-1.66	1	Dehydration
0.58	0.22-2.25	0.72	Use of antibiotics
0.33	0.98-1	0.99	Age
0.59	0.22-2.3	0.73	Leukocytosis
0.72	0.35-2	0.85	Lukopneia
0.72	0.94-4.2	1.98	Bandemia
0.23	0.57-9.5	2.33	Anemia
0.44	0.2-17.7	2.22	Thormbocytosis
0.6	0.1-20.2	1.87	Thrombocytopenia
0.66	0.45-3.4	1.25	CRP
0.33	0.19-1.73	0.58	Hypocalcemia
0.15	0.8-3.7	1.73	WBC in stool

Logistic regression analysis

## Discussion

This study showed that a history of febrile seizure, fever, and hyponatremia are risk factors for seizures in children with shigellosis. Studies in this field are few (4-5, 8-10). Seçmeer et al. have shown that young age and high fever are risk factors for seizure in children with shigellosis, but hyponatremia and the type of Shigella organism are not risk factors. This study was conducted on fifty-five patients with shigellosis, and twenty-nine patients (52.7%) had seizures (8). In this study, although the age of children in the seizure group was significantly lower than the control group, this factor was not identified as a risk factor for seizure. Unlike Seçmeer et al.’s study, in this study, hyponatremia was one of the risk factors for seizure. Afroze et al.’s study on 139 children aged 2-59 months with shigellosis (69 patients with encephalopathy and seventy patients without encephalopathy) has shown that shorter duration of diarrhea before hospitalization, dehydration, sepsis, and hyponatremia are risk factors for exacerbations of disease and death of patients. In this study, encephalopathy is defined as symptoms of seizures, altered consciousness, and coma. In the study of these researchers, S. flexneri was the most common species, and no case of S. dysenteriae has been reported. Furthermore, in this study, the frequency of S.sonnei was higher in the encephalopathy group (5). Similar to Afroze et al.’s study, in the current study, a significant differences was observed between groups regarding the duration of diarrhea before hospitalization, and hyponatremia was considered a risk factor for seizure. Contrary to Afroze et al.’s study, in the present study, 1) No significant difference was found between case and control groups in terms of the severity of dehydration and frequency of Shigella species, 2 ) No case of death, sepsis, and Ekiri syndrome was observed, and 3) S. sonnei was the most common species and we had only one case of S. dysenteriae.Khan et al.’s study on 792 children with shigellosis in Dhaka city has shown that S. flexneri was the most common species with a frequency of 63%, and neurological manifestations were significantly higher in patients with S. dysenteriae type 1 than other species. These researchers reported 10% mortality in their study and mentioned that young age, malnutrition, hyponatremia, low stool frequency, seizure, and coma are the risk factors for death (4). Unlike the study of Khan et al., in this study, firstly, S.sonnei was the most common species of Shigella, and secondly, there was no death among the studied patients. In Chisti et al.’s study, the father’s illiteracy, lack of breastfeeding during the neonatal period, diarrhea with dehydration for less than one day, and growth delay were risk factors for Shigella encephalopathy. This study was conducted on 116 patients under 15 years old with shigellosis (87 patients without encephalopathy and 29 with encephalopathy). These researchers have concluded that educating parents about the value of exclusive breastfeeding and quick correction of dehydration helps to reduce the morbidity and mortality of these patients (9). Shamsizadeh et al.’s study on 154 children with shigellosis has shown that neurological manifestations are present in 68.8% of their cases, and a significant relationship was observed between the occurrence of neurological manifestations and fever and dysentery. In these researchers’ study, there was no significant relationship between the neurological manifestations and other variables such as age, sex, leukocytosis, bandemia, electrolyte disturbances, and type of organism. These researchers have concluded that fever and dysentery are risk factors for neurological manifestations (10). Similar to Shamsizadeh et al.’s study, fever was a risk factor for seizures in this study. The difference in the results of the present studies and the mentioned studies can be due to various factors, including the difference in the selection of study groups, specifically different age groups and types of patients, the type of study (retrospective or prospective), the different distribution of Shigella species and the type of antibiotics resistance pattern. The current study only included Shigella patients with seizure symptoms, while in the mentioned studies (4-5, 9-10), patients with encephalopathy (including seizure patients and various degrees of consciousness) have been evaluated. The lack of death in this study compared to the others studies may be due 1) Low prevalence of underweight and malnutrition in our samples, 2) Quickly and timely treatment of patients, 3) Appropriate response to fluid therapy and antibiotic therapy, 4) Low prevalence of S. dysenteriae type 1, 5) Absence of encephalopathy patients, sepsis and Ekiri syndrome. Fewer cases of ESR more than 30 mm/h, red blood cells more than 10/HPF, bloody diarrhea, and abdominal pain in the seizure group compared to the control group are probably related to the early presentation of seizures, rapid hospitalization, and immediate treatment. Shigellosis is an infectious disease caused by four species of Shigella identified by the type of O antigen and their biochemical properties (11). Shigellosis is still a health problem in the world, primarily in low-and middle-income countries, and it is an essential cause of morbidity and mortality in young children (12). The disease has various clinical manifestations; neurological manifestations are the most common extraintestinal presentation (2). According to the report of Seed et al., 40% of shigellosis patients have neurological manifestations (2). Neurological manifestations can manifest as seizures, headache, lethargy, confusion, neck stiffness, hallucinations, and coma (2, 13).The prevalence of seizure in the studies of Seçmeer et al. (8), Kotloff et al.(1), and Hiranrattana (140 was reported as 52.7%, 5-30%, and 27.6%, respectively. Neurological symptoms, including seizures, may appear before or after the onset of diarrhea (2, 12). Sometimes, seizure presents themselves as Ekiri syndrome. These patients have severe toxicity, convulsions, extreme hyperpyrexia, headache, brain edema without sepsis, or significant dehydration. This syndrome has a fatal outcome (2). The exact pathophysiology of neurological manifestations in shigellosis is still not well known. Considering that infection with negative strains of Shiga toxin can lead to neurological manifestations, it seems likely that other factors apart from the toxin play a role in this relationship (2,6). Sometimes, seizures occur with low fever, which cannot be explained by simple febrile seizures (2). Other reports have mentioned the role of cerebral edema and hemorrhage (5), prostaglandins (15), nitric oxide (16), tumor necrosis factor-alpha (17), interleukin-1beta (17), and neuropeptide corticotropin-releasing hormone (18) in causing seizures.

Limitations of the present study were the need for more access to information about the literacy status of parents and the type of milk consumed in the first two years of life of the studied patients.

## In conclusion

This study showed that a history of febrile seizure, fever, and hyponatremia are risk factors for seizure in childhood shigellosis. Thus, rapid diagnosis and treatment of childhood shigellosis with risk factors is crucial.

## Author’s Contribution

AM, SM conceived the study ,participated in the study design and supervision of project; SO, SE participated in data analysis and software; AM,KH,VC assisted with preparing the document and interpreting the results; ME,SM,BK assisted in data collection; AM ,SM,RD assisted in preparing manuscript and revision. All the authors have read and approved the final submitted manuscript.

## Conflict of interest

The authors have declared no competing or potential conflicts of interest.
